# Heterogeneity in the development of diabetes-related complications: narrative review of the roles of ancestry and geographical determinants

**DOI:** 10.1007/s00125-025-06482-8

**Published:** 2025-07-22

**Authors:** Andrea O. Y. Luk, Yingnan Fan, Baoqi Fan, Edith W. K. Chow, Tony C. K. O

**Affiliations:** 1 Department of Medicine and Therapeutics, The Chinese University of Hong Kong, Hong Kong Special Administrative Region, Shatin, People’s Republic of China; 2 Li Ka Shing Institute of Health Sciences, The Chinese University of Hong Kong, Hong Kong Special Administrative Region, Shatin, People’s Republic of China; 3 Hong Kong Institute of Diabetes and Obesity, The Chinese University of Hong Kong, Hong Kong Special Administrative Region, Shatin, People’s Republic of China; 4 Phase 1 Clinical Trial Centre, The Chinese University of Hong Kong, Hong Kong Special Administrative Region, Shatin, People’s Republic of China

**Keywords:** Ancestry, Diabetes-related complications, Diversity and inclusion, Equity, Ethnicity, Genetics, Geography, Migration, Race, Review, Type 1 diabetes, Type 2 diabetes

## Abstract

The risk of developing diabetes-related complications is influenced by a combination of biological factors, clinical factors and social determinants of health that vary across countries and ethnic groups. Available evidence indicates that the incidence of diabetes-related complications is lower in high-income countries in Europe and North America, while other world regions have higher or variable incidence rates. By ethnicity, White individuals tend to have lower risks of most diabetes-related complications with the exception of coronary artery disease. In contrast, Black, East Asian and South Asian individuals have a greater propensity for most complications, notably chronic kidney disease and stroke. In general, ethnic groups that report a higher incidence of diabetes-related complications also exhibit a higher incidence of type 2 diabetes, particularly among younger age groups. Unique clinical phenotypes characterised by more severe insulin resistance and related comorbidities that drive both an earlier age of diabetes onset and manifestation of end-organ damage have been documented in several high-risk ethnic populations, but the genetic and epigenetic contributors have not been fully elucidated. Non-biological determinants, such as disparities in access to preventive care and the adoption of health-promoting behaviour, shaped by a combination of socioeconomic position, education and culture, are equally as significant as biological factors in influencing disease outcomes, if not more so. In this review we summarise the recent literature on the incidence of diabetes-related complications across countries and ethnic groups, highlighting current gaps in research. We provide perspectives on biological, clinical and social attributes as they relate to geographical and ethnic heterogeneity in the development of these complications. Finally, we discuss the effects of international migration on changing disease trajectories to emphasise the impact of environmental changes on the health of individuals with diabetes.

**Graphical Abstract:**

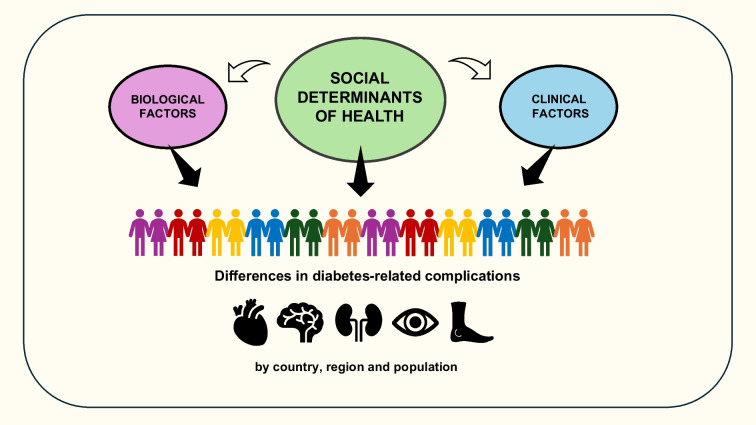

**Supplementary Information:**

The online version contains peer-reviewed but unedited supplementary material including a slideset of the figures for download, available at 10.1007/s00125-025-06482-8.

## Introduction

There is significant heterogeneity in the incidence and prevalence of complications related to diabetes across different regions, countries and ethnicities. Geographical variations in the burden of diabetes-related complications are multifactorial, influenced by differences in socioeconomic development, healthcare resources, environmental exposures, and traditional and cultural practices, along with varying ethnic compositions. There are large inter-ethnic differences in the risks of developing complications even within the same healthcare setting, attributable to disparities in socioeconomic background, the uptake of diabetes interventions and health-promoting behaviours. Phenotypic differences among ethnic groups have been described, which may affect cardiometabolic health and the pattern of complications.

**Fig. 1:**
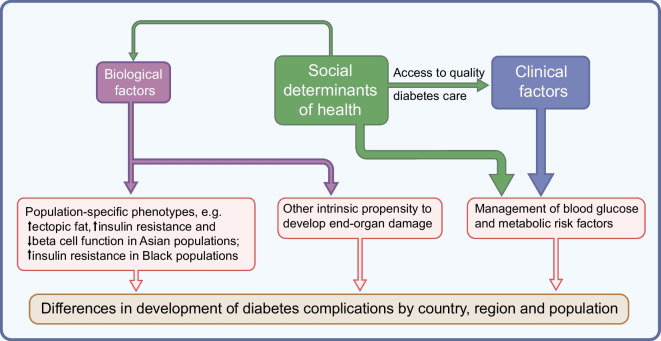
Schematic showing the contributions of biological factors, clinical factors and social determinants of health to heterogeneity in the development of diabetes-related complications across countries, regions and populations. Biological factors, including genetic risk factors, interact with environmental influences to shape phenotype and disease susceptibility. For example, increased visceral adiposity, more severe insulin resistance and impaired beta cell function in Asian populations contribute to an earlier onset of type 2 diabetes and a higher risk of various vascular complications. However, social determinants of health, such as socioeconomic status, culture and traditions, education and health literacy, neighbourhood and food security, along with clinical factors, such as disease monitoring, access to effective drugs and technologies, adoption of health-promoting behaviours and psychosocial stress, affect the management of blood glucose and metabolic risk factors and are more significant than biological factors in driving differences in incidence of diabetes-related complications between populations. This figure is available as part of a downloadable slideset

We searched the published literature from 1 January 2015 to 31 December 2024 to identify studies on the incidence of diabetes-related complications across different countries and regions, as well as across different ethnic groups within the same country (see electronic supplementary material [ESM] Tables 1 and 2). For studies involving Indigenous populations and ethnic disparities in microvascular complications, the search period was extended to start from 1 January 1990 because of the scarcity of contemporary studies. This review focuses primarily on populations with type 2 diabetes, for which more evidence is available; ethnic differences in complications in type 1 diabetes are examined in a separate section. It is important to recognise that variations in study design and methodology used to assess diabetes-related complications pose substantial challenges in understanding their extent across countries and regions, complicating meaningful comparisons. Furthermore, the lack of consensus on the use and reporting of race and ethnicity as population descriptors in healthcare research requires caution in interpretation of the results, as there is a tendency to ascribe observed differences to biological factors without considering the social and cultural dimensions inherent in these constructs. In this review we discuss the possible biological and clinical factors, in addition to social determinants of health, that drive geographical and ethnic diversity in the development of diabetes-related complications (Fig. 1). We address the influence of migration to highlight the impact of changes in lifestyle, environment and healthcare on disease outcomes. We also summarise the available genome-wide association studies (GWAS) related to complications and reference key studies that report ancestry-specific genetic loci (see ESM Table 3 for search strategy).

## Heterogeneity in incidence of diabetes-related complications by country or region

### Methodological variations and limitations in studies on complications

Most recent studies reporting the incidence of diabetes-related complications have been conducted in Europe [1, 26], North America and Oceania [27, 30] and the Western Pacific [31, 61]. There is a paucity of large population-based studies conducted in Africa [62, 64], the Middle East and North Africa [65, 71], South and Central America [72, 73] and South-East Asia [74, 75], where diabetes prevalence is highest. In these regions, we identified studies using mostly hospital-based cohorts with smaller sample sizes, which may not be representative of the local diabetes populations.

The documented incidence and prevalence of clinically silent complications such as kidney and eye diseases are further influenced by screening practices, which vary across healthcare systems. Standardised measurements and reporting, particularly for microvascular complications, are lacking, and definitions differ across studies. For instance, studies have used albuminuria, eGFR and diagnostic codes, individually or in combination, to define diabetic kidney disease. Likewise, there is a lack of uniformity in the definition of CVD, which may encompass various outcomes, including myocardial infarction, acute coronary syndrome, revascularisation procedures, stroke and the terminal outcome of cardiovascular death. Agreement is needed on the most appropriate methods for identifying and defining major complications that are also feasible in resource-limited settings.

### Low incidence of CVD and diabetic kidney disease in Europe, North America and English-speaking countries in the Western Pacific

**Fig. 2:**
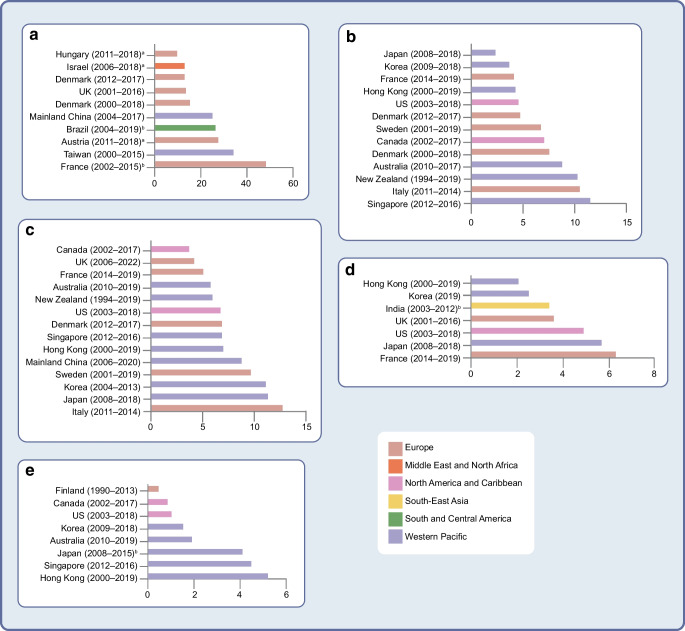
Incidence rates (per 1000 person-years) of major adverse cardiovascular events (**a**), myocardial infarction (**b**), stroke (**c**), peripheral artery disease (**d**) and end-stage kidney disease (**e**) in individuals with type 2 diabetes across different regions and countries from studies published between 2015 and 2025. Different colours represent different world regions. ^a^Clinic-based cohort studies; ^b^hospital-based cohort studies; all other studies are population-based cohort studies. This figure is available as part of a downloadable slideset

Our review of the recent literature on diabetes-related complications by country and region revealed several key findings. High-income countries in Europe and North America and English-speaking nations in the Western Pacific consistently report the lowest incidence rates for most diabetes-related complications. For example, Denmark [24], Hungary [1] and the UK [13] have among the lowest rates of major adverse cardiovascular events, which comprise non-fatal myocardial infarction, non-fatal stroke and cardiovascular death (Fig. 2, ESM Table 4). Additionally, the incidence rates of end-stage kidney disease are lower in Australia [31], Canada [27], Finland [17] and the USA [28] than in the Western Pacific (Fig. 2 ESM Table 5).

High-income countries generally have more robust healthcare infrastructure that supports regular screening, early detection and prevention of complications. Studies conducted in populations with diabetes in high-income countries such as Australia, Denmark, the UK and the USA over the past two decades show that there has been a decline in the incidence of CVD and kidney disease [76, 79]. An analysis of all-cause mortality data from 19 data sources in high-income regions found that age- and sex-standardised mortality rates decreased in 17 of these sources [80]. Improvements in diabetes care models, increased uptake of organ-protective medications and implementation of effective public health policies largely account for these observations. In contrast, in low- and middle-income countries, such as India, there has been a notable increase in mortality rates from coronary heart disease and stroke, particularly in rural areas [81]. These divergent trends may further exacerbate global disparities in health outcomes for individuals with diabetes.

### High incidence of diabetic kidney disease in East Asia

East Asia has low rates of CVD but high rates of diabetic kidney disease (Fig. 2, ESM Tables 4 and 5). The incidence rates of myocardial infarction in Hong Kong, Japan and South Korea are comparable to those reported in Europe and North America [34, 36]. Conversely, the incidence rates of end-stage kidney disease appear to be elevated, with rates of 5.3 per 1000 person-years (PY) reported in Hong Kong [34], 4.1 per 1000 PY reported in Japan [43] and 4.4 per 1000 PY reported in Singapore [38]. A review of the worldwide epidemiology of diabetes-related end-stage kidney disease estimated a threefold higher incidence in the Western Pacific than in Europe, with these regions having the fastest and slowest annual increases, respectively [82]. Multiethnic cohort studies have consistently shown that Asian ethnicity is associated with higher odds of albuminuria than White ethnicity, suggesting possible aetiopathological differences [83, 84]. Furthermore, the progression to end-stage kidney disease is linked to population longevity; in this context, Hong Kong, Japan and Singapore rank among the top ten jurisdictions with the longest life expectancies.

### Incidence of diabetic retinopathy by region

The significant variability in the methodologies used for identifying complications has rendered it difficult to derive insights into the comparative incidence and prevalence of diabetic retinopathy and neuropathy across countries (ESM Table 6). The use of disease coding often underestimates the burden of complications, which have a prolonged subclinical phase, with only the most advanced stages recorded. In China, a hospital-based study reported an incidence rate of diabetic retinopathy of 79.6 per 1000 PY using systematic fundus photography [48], while a population-based study using ICD-10 coding reported a much lower rate of 2.9 per 1000 PY [32]. The IDF recently presented data on the global prevalence of diabetic retinopathy, revealing regional disparities, especially for sight-threatening diabetic retinopathy, with the highest prevalences estimated in North America and the Caribbean and the Middle East and North Africa [85].

### Incidence of diabetic neuropathy by region

Diabetic neuropathy is a complication with the least consensus in diagnosis, as various scoring systems that combine symptoms and signs have been employed (ESM Table 7). The subjective nature of symptom reporting and variability in detecting physical signs, such as sensory deficits and diminished ankle reflexes, complicate assessment. Clinic- or hospital-based cohort studies that identified diabetic neuropathy based on symptoms and/or lower limb examinations [49, 51, 70, 72, 74, 75], reported incidence rates ranging from 21.4 per 1000 PY in Ethiopia [70] to 130.2 per 1000 PY in Pakistan [75]. The methodological variability among studies is likely to overshadow any regional differences, thereby masking the true variations in the prevalence and incidence of diabetic neuropathy across regions, should such variations exist. There appears to be fewer disparities in incidence rates of lower extremity amputation across countries [22, 23, 27, 28, 31, 46, 53, 56].

### Limited studies on complications outside Europe, North America and the Western Pacific

Regions with the highest prevalence of or number of individuals living with type 2 diabetes, including the Middle East and North Africa, South and Central America and South-East Asia, are under-represented in population-based studies on diabetes-related complications [85]. Most studies from these regions have recorded very high incidence rates of complications, including CVD, diabetic kidney disease and advanced diabetic retinopathy. However, many of these cohorts were recruited from hospital settings [62, 67, 72, 75], which may lead to inflated estimates due to the inclusion of individuals with more advanced diabetes. Systematic screening for complications and data collection are essential to quantify the overall global impact and burden of diabetes; however, effective surveillance systems are absent in many regions.

Disparities in the incidence of diabetes-related complications across regions may be attributed to biological differences in predisposition to certain complications among some populations, such as the higher incidence of kidney disease in East Asian individuals. However, non-biological factors, including socioeconomic status, healthcare systems, cultural practices and physical environment, are likely to play a major role [86, 87]. Low- and middle-income countries face significant challenges, including insufficient healthcare capacity, inadequate training of healthcare workers in the management of diabetes and limited access to regular monitoring and treatment; in addition, there may be a lack of guidelines tailored to the local context to support effective clinical management [88, 89]. A multicountry study on the availability and affordability of essential medications for diabetes found that metformin was available in fewer than two-thirds of the pharmacies surveyed, while insulin was accessible in only 10% of pharmacies in low-income countries [90]. In these regions, approximately one-quarter of households could not afford metformin and over 60% could not afford insulin [90]. In certain African nations, healthcare resources are prioritised for the management of communicable diseases such as HIV/AIDS and hepatitis B and C, despite their strong links with type 2 diabetes, chronic kidney disease and CVD [89, 91]. Local cultures, traditions and beliefs significantly influence the adoption of healthy lifestyles and health-seeking behaviours, impacting the control of risk factors and outcomes. A strong reliance on traditional medicine in certain countries may discourage individuals from engaging with Western healthcare services and divert financial resources away from evidence-based healthcare practices. Consequently, glycaemic regulation and management of cardiometabolic risk factors are often suboptimal in low- and middle-income countries, with studies showing that only 30% of individuals achieve the HbA_1c_ target of <7.0% [92, 93], compared with up to 50% in high-income countries [94].

## Heterogeneity in incidence of diabetes-related complications by ethnicity

### Defining race, ethnicity and ancestry

In medical research, race and ethnicity are frequently used interchangeably to infer ancestry. Most studies on diabetes-related complications among different racial and ethnic groups have been conducted in settler colonial states and high-income countries, including the USA, Canada, Australia, New Zealand and the UK, and in multiethnic communities such as Singapore. The concepts of race and ethnicity arise from observed differences in physical appearance, cultural practices, religion, linguistics and geopolitical factors between groups of people (see Text box: Definitions used in this review). These labels are rooted in the notion that people share similar traits based on their geographical origins. Yet, racial and ethnic groupings can be arbitrary and lack biological basis, as shown by studies revealing discordant genetic ancestral markers [95, 96]. Moreover, assigning ethnic or racial categories may falsely assume homogeneity among individuals included within the groups. For instance, the term ‘Hispanic’ is used to describe Spanish-speaking people living in the USA, which encompasses individuals with origins in Central and South America as well as Spain. The term ‘Asian’ collectively represents individuals from over 40 countries. Lastly, classifications of racial and ethnic categories used in the medical literature are partly shaped by social and legal legislature, which may change over time. Thus, our current report on racial or ethnic disparities among individuals with diabetes relies on how investigators classified their study participants, which may obscure meaningful differences within subpopulations. We acknowledge these limitations; however, to remain true to the reported study findings, we present results using population descriptors presented in the original studies.


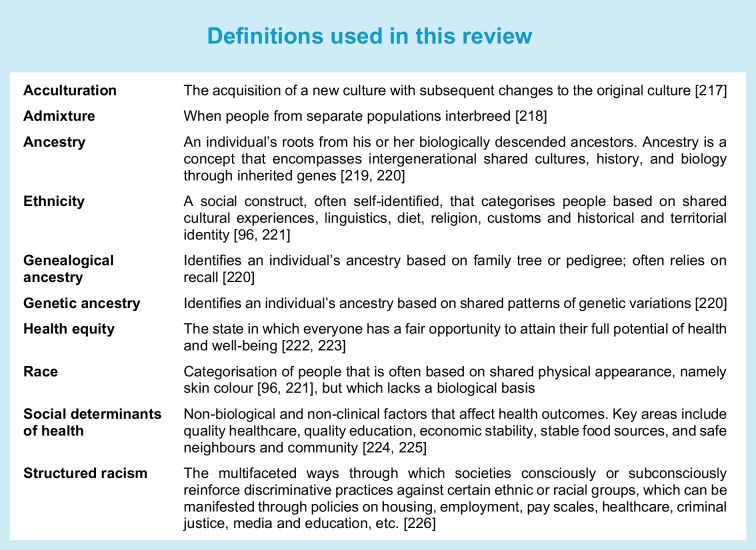


### Higher incidence of diabetic kidney disease in non-White ethnic groups

Diabetes-related complications, especially microvascular complications, disproportionately affect minority ethnic populations compared with White population [97]. For instance, albuminuria, an early sign of chronic kidney disease, occurs more frequently among minority ethnic groups. In cross-sectional studies, the odds of albuminuria were increased 1.4- to 4-fold in Asian populations (including East Asian, South Asian and Pacific Islander populations), 1.3- to 1.4-folds in Hispanic individuals and 1.5-fold in non-Hispanic Black individuals compared with White populations when adjusted for glycaemic parameters, blood pressure, use of renin–angiotensin–aldosterone inhibitors and history of CVD [83, 84]. It is noteworthy that ethnic groups experiencing a higher risk of microvascular complications have an earlier age of onset of type 2 diabetes, which is associated with a worse glycaemic trajectory that drives damage to the microvasculature and end organs. In this context, South Asian individuals have greater amounts of visceral fat than Latino, White, African and other Asian subgroups [98], while African American and Hispanic individuals have a higher BMI and more severe insulin resistance than White individuals [99, 100]. The ‘Asian phenotype’, characterised by pancreatic beta cell defects, increased levels of ectopic fat and more severe insulin resistance with a normal BMI, is regarded as the main pathophysiological basis for the higher incidence of type 2 diabetes, including young-onset type 2 diabetes, in this population [101]. Insulin resistance has widespread metabolic consequences, including hypertension, dyslipidaemia, elevated proinflammatory and prothrombotic markers and endothelial dysfunction, which increases the risks for chronic kidney disease and atherosclerosis.

Epidemiological studies have shown a higher burden of end-stage kidney disease among non-White ethnic groups, with or without diabetes [102, 103]. Research indicates that 40% of African American individuals with chronic kidney disease without diabetes have non-linear declines in GFR [104], similar to findings in individuals of African ethnicity with diabetes, who have tenfold higher adjusted odds of a stable decline in renal function followed by an accelerating eGFR decrease than those with non-African ethnicity [105]. The increased prevalence of hypertension and lower achievement of blood pressure targets in African populations may exacerbate the progression of kidney diseases [106]. However, access to care, including access to dialysis centres, and the quality of care provided are often unmeasured and significantly confound outcomes [103]. Uninsured individuals with chronic kidney disease who are not on dialysis are less likely to receive optimal medical therapy and are thereby more susceptible to the progression of cardiometabolic risk factors [107]. Nonetheless, among insured individuals, despite receiving comparable care [108], minority ethnic groups continue to have 1.5- to 2.0-fold higher risks for end-stage kidney disease than their White counterparts [109].

### Incidence and prevalence of diabetic neuropathy and amputation by ethnicity

The reported prevalence of diabetic neuropathy among minority ethnic populations is generally similar to or lower than that among White individuals [110, 113]. In the USA, African American and Hispanic individuals are more likely than White individuals to undergo lower extremity amputations [114, 116], which may be related to lower rates of revascularisation in these groups [117]. Conversely, studies from the UK have not consistently shown a higher incidence of amputation among individuals of African descent [118]. South Asian individuals, mainly Indian Asian individuals, have a lower risk of foot ulcers and amputation secondary to diabetes than White individuals [54, 119, 121]. Studies have shown that South Asian individuals have better preserved peripheral nerve quality, which may protect against the development of foot ulcers [112]. A study from the UK using objective electrophysiological nerve assessments revealed that Indian Asian individuals had better preserved large and small fibre function than White individuals when controlled for age, diabetes duration, smoking and peripheral blood flow indicators [122].

### Incidence of CVD by ethnicity

There are discordant association patterns between minority ethnicities and the risks of coronary artery disease, peripheral artery disease and stroke. A tower incidence of coronary heart disease and peripheral artery disease, coupled with a higher incidence of stroke, has been observed in East Asian and Black individuals than in White individuals, while South Asian individuals with diabetes show a lower risk of peripheral artery disease but higher risks of coronary heart disease and stroke than White individuals with diabetes [120, 123]. These differential risks of CVD subtypes by ethnicity have been noted in both observational cohorts and clinical trial settings, where similar levels of care are provided to all participants. Among those enrolled into the Action in Diabetes and Vascular Disease (ADVANCE) study, participants from Asia had a statistically significantly lower incidence of coronary heart disease and peripheral artery disease but higher rates of stroke than participants from Australasia, North America and Western Europe combined [124]. The reasons for the increased incidence of stroke in certain populations are unclear, but differences in blood pressure and other factors such as impaired cerebral autoregulation may play a role [125]. Additionally, the risk association between systolic blood pressure and incident stroke is stronger in Black and South Asian populations than in White populations, indicating greater sensitivity to blood pressure effects in these groups [126, 127].

Notably, there is heterogeneity in CVD outcomes among Asian subgroups [123, 128, 132], with South Asian groups, particularly Malay and Indian populations, demonstrating overall higher risks for atherosclerosis than other Asian communities. Nonetheless, Indian Asian individuals, despite having a worse cardiometabolic risk factor profile, show less lower limb ischaemia, as measured by femoral intima–media thickness, while having a similar coronary calcium score to White individuals [133]. The lower prevalence of smoking and alcohol consumption among Indian Asian individuals cannot fully explain these patterns and further investigation into other biomedical factors is needed.

### Influence of social determinants of health on risks of diabetes-related complications

In settler colonial states, severe disparities in health standards between Indigenous and non-Indigenous populations have been documented [134]. Some of the earliest evidence came from studies of Akimel O’odham people (previously referred to in the literature as ‘Pima Indians’) in Arizona, USA, which described an earlier onset of diabetes and accelerated progression to end-stage kidney disease [135, 136]. Similarly, it has been reported that American Indian people with diabetes have a higher prevalence of microvascular complications and stroke but a lower prevalence of coronary artery disease than US adults with diabetes [137], and Māori and Pacific Islander peoples from New Zealand and Indigenous Australian individuals with diabetes have higher odds or hazards of albuminuria [138], end-stage kidney disease [37], severe retinopathy [139], lower limb amputation [54] and CVD [140] than non-Indigenous groups with diabetes.

While associations between ethnicity and the risk of diabetes-related complications may suggest a degree of biological susceptibility, they more prominently highlight disparities in healthcare access and social determinants of health. Multiple risk factor management can help prevent cardiovascular and kidney complications in individuals with type 2 diabetes but this is less likely to be achieved in minority ethnic populations. Despite global efforts to address health inequities [141, 143], unconscious bias among healthcare providers may exacerbate inequalities in prescribing practices, diabetes education and the provision of other diabetes support. Studies in the UK have reported that Black and Asian individuals have higher HbA_1c_ levels, attributed to less frequent laboratory monitoring and delays in intensifying insulin and non-insulin glucose-lowering therapies [144, 145]. Among participants in the Look AHEAD study, Black and American Indian individuals were less likely to initiate newer classes of glucose-lowering medications than their White counterparts [146]. Ethnic disparities in diabetes screening have also been illustrated in a study in the USA, which showed a higher likelihood of undiagnosed diabetes among Asian, Black and Hispanic individuals than among White individuals [147]. Delaying diabetes diagnosis prolongs untreated hyperglycaemia, which adds to the risk of end-organ damage.

Numerous studies indicate that, even after adjusting for clinical factors, residual ethnic differences persist in the risks of diabetes-related complications [111]. Low socioeconomic status has been shown to correlate with a higher prevalence of diabetic kidney disease and retinopathy, which may confound the association between ethnicity and complication risks [97]. Minority ethnic groups often congregate in geographical areas with limited access to healthcare facilities. In the context of diabetes management, inadequate access to healthy food and safe spaces for regular physical exercise can adversely affect disease control [148, 150]. Low health literacy, partly due to limited language proficiency among certain minority ethnic and Indigenous populations, poses major barriers to navigating healthcare systems and effectively communicating with healthcare providers [151, 152]. Differing beliefs about perceived health status [153] and the use of alternative medicine [154, 155] may also influence health-seeking behaviours [156], screening for complications [157, 160] and adherence to therapy [161, 162]. Psychosocial stress arising from poverty, employment instability, traumatic experiences and discrimination can have a negative effect on diabetes self-management and outcomes. Perceived racial discrimination is associated with higher HbA_1c_ levels [163], which may be mediated by increased levels of diabetes distress [164].

## Correlation of ancestry with genetic predisposition to diabetes-related complications

### Limited GWAS in non-European populations

Geographical and ancestral heterogeneity may stem from genetic differences. Challenges in the interpretation of GWAS of diabetes-related complications partly arise from inconsistent phenotypic definitions across studies and insufficient data from non-European populations, including Indigenous populations, hindering the detection of robust genetic signals. Furthermore, there are increasing numbers of individuals with genetic admixture, influenced by the recent history of colonialism, as observed in countries such as the USA, and increases in international migration. Admixture mapping may offer advantages over traditional association mapping, particularly for discovering significant signals in recently mixed populations, such as Hispanic or Latino groups [165, 167]. Interactions among genetic, metabolic, lifestyle, environmental and psychosocial factors and prenatal exposures may further contribute to the heterogeneity in complication development through epigenetic changes. Although our current understanding of the genetic contributions to ancestry-related heterogeneity in diabetes-related complications is incomplete, advancements in technical and statistical methodologies present promising prospects for the future.

Single-ancestry GWAS on kidney diseases in type 2 diabetes have identified the *FTO* locus in Japanese individuals, *OR5V1* and *HIATL1* in Korean individuals and *RND3*/*RBM43*, *SLITRK3*, *ENPP7*, *GNG7* and *APOL1* in African American individuals, among others [168, 170]. The Family Investigation of Nephropathy and Diabetes (FIND) consortium conducted a GWAS on advanced diabetic kidney disease in multiethnic groups, identifying SNPs between the *SCAF8* and *CNKSR3* genes both in American Indian individuals and in a trans-ethnic meta-analysis [171]. The Surrogate Markers for Micro- and Macrovascular Hard Endpoints for Innovative Diabetes Tools (SUMMIT) consortium reported a lead SNP near *GABRR1* associated with microalbuminuria in European individuals but not in Asian individuals, while the *UMOD* and *PRKAG2* loci were associated with GFR in type 2 diabetes in both European and Asian individuals [172]. Most trans-ethnic studies did not show significant ancestry-correlated heterogeneity in the effects of the top-ranked signals.

Genetic studies examining diabetic retinopathy and neuropathy are scarce. An early GWAS of Taiwanese Chinese individuals discovered five loci at *MYSM1*, *PLXDC2*, *ARHGAP22* and *HS6ST3* associated with diabetic retinopathy, with some of the genes being linked to endothelial cell angiogenesis and capillary permeability [173]. A SNP in the *NVL* gene was found in a GWAS on proliferative diabetic retinopathy in European individuals, but not in trans-ethnic meta-analysis [174]. The *GRB2* gene locus, encoding an EGF receptor-binding protein in the retina, was significant for sight-threatening diabetic retinopathy in a meta-analysis combining individuals with type 1 and type 2 diabetes from European and Indian ancestries [175].

### Genetic architecture in specific populations

Geographically isolated populations, such as the Inuit people of Greenland, display distinct genetic architectures compared with other populations. This may be linked to genetic shifts following population bottlenecks and adaptations to natural selection, influenced by limited migration rates, isolation and a restricted diet. In this population, 50% of common variants associated with metabolic traits are Arctic-specific, including a type 2 diabetes variant at *TBC1D4* related to skeletal muscle resistance [176]. This suggests an adaptation to the Inuit people’s low-carbohydrate diet. Homozygous carriers of this common nonsense variant demonstrated a tenfold increased risk of type 2 diabetes and explained 15% of the occurrence of type 2 diabetes in Greenland [177]. Similarly, loci indicating adaptation to environmental and dietary factors in isolated populations include *FADS1–3*, which influence polyunsaturated fatty acid metabolism in Inuit people, and *APOC3*, which is linked to cardioprotection in Greek and Amish populations [178, 180]. The unique characteristics of isolated populations provide opportunities to enhance the power to detect low-frequency genetic variations for complex diseases under more homogeneous environmental exposures.

### Ancestry- and pathway-specific polygenic risk scores for type 2 diabetes and complications

Clustering analysis has been used to partition common type 2 diabetes variants into clusters that represent different physiological pathways, which may be differentially associated with end-organ complications [181]. In a large multi-ancestry GWAS comprising over 2.5 million individuals (40% non-European), ancestry-specific differences in the allelic effects of index SNPs within each cluster were observed. The allelic effects of SNPs in the beta cell dysfunction cluster were stronger in East Asian individuals, whereas those in the lipodystrophy and obesity clusters were greater in European individuals [182]. This study further detected positive associations of the lipodystrophy and obesity clusters with coronary heart disease and the obesity cluster with kidney and eye complications, and a negative association of the beta cell dysfunction cluster with kidney disease [182]. However, no obvious ancestry heterogeneity was observed in the associations between cluster-partitioned polygenic risk scores and complications [182].

## Acculturation and effect of migration on risk of diabetes-related complications

Acculturation is the process by which non-dominant populations adopt the values, attitudes and behaviours of the dominant group, thereby integrating their cultures [183]. Studies have reported worsening cardiometabolic health outcomes associated with acculturation, which may be related to the adoption of a ‘Western’ lifestyle [184]. Conversely, acculturation may improve health outcomes for migrants by increasing their uptake of health-seeking behaviours and use of healthcare services [185]. Research comparing the risks of diabetes-related complications among individuals belonging to the same ethnic group but residing in different locations provides valuable insights into the significance of non-biological factors for health outcomes. Several studies have examined the effects of migration to the USA on the metabolic and vascular health of Japanese individuals. One study compared metabolic indices and carotid intima–media complex thickness between a Japanese American population and a Japanese population, both without diabetes, and found that the Japanese American individuals showed more rapid progression in carotid intima–media thickness, accompanied by a higher BMI and higher blood lipid and blood glucose levels, than their Japanese counterparts [186]. Additionally, another study found that the prevalence of coronary artery disease and proteinuria was higher among Japanese American individuals with diabetes than among Japanese individuals with diabetes, whereas the prevalence of diabetic retinopathy was similar between the two populations [187].

Long-term follow-up of first-generation migrants and cross-generational migrants enables the assessment of potential dose- or time-dependent effects of acculturation. An earlier study on the influence of Westernisation on progression of atherosclerosis revealed that the consumption of animal fats and simple carbohydrates increased and physical exercise levels decreased in the order of Japanese individuals followed by first-generation, second-generation and later generations of Japanese American individuals [188]. However, a more recent study suggested a narrowing or reversal of the gap over time between Japanese American and Japanese individuals across multiple metabolic parameters [189]. Similarly, Indian people residing in Singapore are reported to have a higher prevalence of diabetic retinopathy than those residing in urban India (30% vs 18%) [190, 192]. Longer residence in Singapore was associated with an increased risk of diabetic retinopathy among first-generation Indian migrants with diabetes, while second-generation migrants have an additional 73% higher odds of developing diabetic retinopathy than their first-generation counterparts [193].

Collectively, these studies suggest that migration can adversely influence metabolic health and increase the risk of complications in people with diabetes. Individuals forced to migrate to escape persecution, war, natural disasters or economic crises are likely to be the most vulnerable, as they may lack access to mainstream healthcare and their health needs may not be fully met because of communication barriers. However, studies specifically focusing on refugees are currently lacking.

## Ethnic differences in diabetes-related complications in type 1 diabetes

Many studies have reported that Black or African individuals with type 1 diabetes have a higher risk of microvascular complications than White individuals with type 1 diabetes. In a longitudinal cohort of people with type 1 diabetes in the UK, African Caribbean individuals experienced a more rapid decline in eGFR than White individuals, with a 1.6-fold higher risk of progressing to advanced kidney disease [194]. Conversely, a Brazilian study on type 1 diabetes found no correlation between self-reported ethnicity or African genomic ancestry and the presence of chronic kidney disease [195]. In a single-centre study in South Africa, the age- and sex-adjusted odds of any diabetic retinopathy and referrable diabetic retinopathy were 1.7-fold and 3.4-fold higher, respectively, in African vs White individuals with type 1 diabetes [196]. A cross-sectional cohort study in the UK found that African Caribbean ethnicity was independently associated with a 1.4-fold increased hazard of sight-threatening diabetic retinopathy compared with non-African Caribbean ethnicity [197]. Another population-based study in the UK indicated that diabetic retinopathy of any severity was more prevalent among White individuals, whereas severe diabetic retinopathy was more common in Black individuals [198]. Besides potential differences in risk factor management, the consistent observations that African or Black individuals are more likely to have advanced ophthalmic complications may arise because of less frequent eye assessments in this group, resulting in missed opportunities for early intervention [199]. Several studies have investigated the comparative risks of diabetic retinopathy between Asian and White individuals with type 1 diabetes, yielding conflicting findings [196, 200]. Ethnic differences in diabetic neuropathy are reported less frequently. However, a US-based study showed that Black ethnicity was associated with a twofold increased odds of diabetic peripheral neuropathy than White ethnicity when assessed using a validated questionnaire [201].

Hospitalisation for CVD in individuals with type 1 diabetes was examined in a population-based study in the UK, which showed that Black individuals were 40% less likely to be admitted for myocardial infarction than White individuals, whereas Asian individuals had a 20–80% increased risk of myocardial infarction or stroke [202]. Several studies have assessed the early stages of atherosclerosis in individuals with type 1 diabetes using non-invasive measurements [203, 205]. Among participants with type 1 diabetes in the SEARCH for Diabetes in Youth study, carotid–femoral pulse wave velocity, an indicator of arterial stiffness, was higher in non-Hispanic Black individuals than in their White counterparts [205].

There are also distinct differences in glycaemic trajectories and metabolic attributes across ethnic groups. Several studies have shown that non-White ethnic groups, including Black, Hispanic, Asian and Indigenous populations, have higher HbA_1c_ values at diagnosis of type 1 diabetes, and this difference persists for years following diagnosis [206, 210]. The under-use of diabetes technologies among minority ethnic groups, such as continuous glucose monitoring and insulin pumps and intensive insulin regimens, has been well documented [211, 213]. It has been shown that inequities in treatment intensity explain approximately one-third of the difference in HbA_1c_ values between White and non-White ethnic groups and more than half of the excess risk of microvascular complications in non-White groups [214]. Similar to type 2 diabetes, evidence also suggests that Black, Hispanic, Asian and Indigenous individuals with type 1 diabetes experience more severe insulin resistance [206, 208, 210, 215], which contributes to higher HbA_1c_ values and insulin requirements.

## Implications and future research

In this review, we have collated recent literature on the local incidence of diabetes-related complications across different countries and regions, summarising studies that compare the prevalence and incidence of complications by ethnic groups. We identified several gaps in the existing literature (Table 1). First, there is a paucity of population-based studies on diabetes-related complications from the Middle East and North Africa, South-East Asia, and South and Central America, regions that also have the highest diabetes prevalence. In some of these regions, diabetes remains under-prioritised with regard to healthcare resource allocation. Quantifying the health and economic burden of diabetes against the burdens of other communicable and non-communicable diseases is informative for public health planning and may catalyse system change, leading to the inclusion of diabetes care as one of the essential mandates of healthcare systems. Second, differences in participant selection and diagnostic criteria for complications make it difficult to draw definitive conclusions regarding regional variations in complications. The lack of uniformity in the assessment and definition of microvascular complications, particularly diabetic retinopathy and neuropathy, is problematic, as clinical assessments are often subjected to interobserver variability. Improved alignment in the reporting of diabetes-related complications is necessary to facilitate meaningful comparisons across regions and populations. Third, ideas of race and ethnicity are deeply intertwined with a complex history of colonialism, racism and discrimination. Recent advancements in genetic research have arisen as possible solutions to the use of these population identifiers when assessing disease heterogeneity, yet most current genetic analyses focus on individuals of European ancestry. These concepts of race and ethnicity are informed by self-identity, shared cultural experiences and interactions with other social determinants of health. However, these aspects are often overlooked in scientific reporting, which tends to attribute health disparities to biological differences. In the continually evolving field of genetic and precision medicine, researchers are encouraged to consult current guidelines on the appropriate use and reporting of population descriptors within the context of their health research. Fourth, unique disease phenotypes have been described for various populations; however, more research is necessary to understand the genetic and epigenetic underpinnings of this diversity. Future genetic studies should broaden their inclusion criteria to encompass individuals of non-European ancestry and admixed populations. Finally, there is a lack of well-conducted studies examining the impact of migration on health outcomes in individuals with diabetes. In 2020, over 280 million people, or 3.6% of the world’s population, lived outside their country of birth, representing an increase of approximately 80% since 1990 [216]. Given this trend of a growing global migrant population, studies are needed to document the health status of and address care gaps within this demographic. Such research will provide insights into how changes in the physical environment, such as changes in climate and exposure to environmental pollutants, may influence disease progression.

**Table 1:** Summary of gaps in the literature on the heterogeneity of diabetes-related complications by population and actions needed

Gap in literature	Considerations and action
Lack of population-based studies on diabetes-related complications in low- and middle-income countries	There is a need for effective surveillance and data collection to quantify the burden of diabetes and its complications
Variations in diagnostic criteria between studies; inconsistencies in assessing and reporting microvascular complications are especially problematic	Uniformity in definitions and reporting of complications is needed for meaningful comparisons across regions; methods used should be feasible in resource-limited settings
Lack of agreement on the use and reporting of race and ethnicity as population descriptors in healthcare research	Review and consult current guidelines on the use and reporting of population descriptors that are appropriate for the objectives and context of the research being carried out
Unique disease phenotypes exist in different populations, but more research is needed to understand the genetic and epigenetic factors involved	Future genetic studies should include non-European and admixed populations
Lack of studies on the impact of international migration on health outcomes for individuals with diabetes	Research is needed to document health status and care gaps in this population to provide insights into how environmental changes may affect disease progression

## Conclusion

The evidence indicates that low incidences of diabetes-related complications are observed in high-income countries in Europe, North America and some parts of Asia, while relatively higher incidences are found in other regions. However, the lack of population-based studies in numerous parts of the world makes it difficult to appreciate the full pattern of geographical variations. Ethnic groups with a higher incidence of type 2 diabetes also demonstrate increased risks of microvascular complications and varying risks of CVD. Non-biological factors may be more significant drivers of the observed regional and ethnic heterogeneity than biological attributes. Globalisation and medical advancements may widen these differences, as individuals with access to new technologies may experience improved health outcomes, while the health status of those less privileged may remain stagnant or deteriorate over time. The development and systematic evaluation of contextually relevant and culturally tailored disease management programmes in low- and middle-income countries and among minority ethnic populations are needed to improve equity in diabetes care delivery.

## Supplementary Information

Below is the link to the electronic supplementary material.

ESM Tables (PDF 367 KB)

Slideset of figures (PPTX 313 KB)

## Abbreviations

GWAS: Genome-wide association studies
PY: Person-years
