# Supplementary material for: Heterogeneity in the development of diabetes-related complications: narrative review of the roles of ancestry and geographical determinants
Source: Diabetologia. 2025 Jul 22;68(11):2386–404. doi: 10.1007/s00125-025-06482-8 (PMC12534336; doi:10.1007/s00125-025-06482-8)
Supplement: Supplementary file 1 — ESM Tables (PDF 367 KB) [file 125_2025_6482_MOESM1_ESM.pdf]

Heterogeneity in progression of diabetes-related complications – role of ancestry and geographical determinants

ESM Table 1. Search strategies of cohort studies reporting incidence of diabetes-related complications in people with type 2 diabetes across different regions and countries.

ESM Table 2. Search strategies of cohort studies reporting incidence or prevalence of diabetes-related complications in people with type 2 diabetes across different ethnic or racial groups

ESM Table 3. Search strategies of genetic studies for diabetes-related complications in type 2 diabetes groups

ESM Table 4. Details of studies reporting incidence rate (per 1,000 person-years) of cardiovascular disease in people with type 2 diabetes across different regions and countries.

ESM Table 5. Details of studies reporting incidence rate (per 1,000 person-years) of chronic kidney disease and end-stage kidney disease in people with type 2 diabetes across different regions and countries.

ESM Table 6. Details of studies reporting incidence rate (per 1,000 person-years) of diabetic retinopathy in people with type 2 diabetes across different regions and countries.

ESM Table 7. Details of studies reporting incidence rate (per 1,000 person-years) of diabetic neuropathy and non-traumatic lower limb extremity amputation in people with type 2 diabetes across different regions and countries.

ESM Table 1. Search strategies of cohort studies reporting incidence of diabetes-related complications in people with type 2 diabetes across different regions and countries.

Step	Search terms in PubMed
1	(diabetes mellitus, type 2[MeSH Terms]) OR (type 2 diabetes[Title/Abstract])
2	(Cardiovascular Diseases[MeSH Terms]) OR (cardiovascular disease[Title/Abstract]) OR (coronary artery disease[Title/Abstract]) OR (myocardial infarction[Title/Abstract]) OR (stroke[Title/Abstract]) OR (peripheral artery disease[Title/Abstract]) OR (amputation[Title/Abstract])
3	(Diabetic Nephropathies [Mesh Terms]) OR (Renal Insufficiency, Chronic[MeSH Terms]) OR (chronic kidney disease[Title/Abstract]) OR (end-stage kidney disease[Title/Abstract]) OR (kidney failure[Title/Abstract]) OR (albuminuria[MeSH Terms]) OR (albuminuria[Title/Abstract])
4	(Diabetic Retinopathy[MeSH Terms]) OR (retinopathy[Title/Abstract])
5	(Diabetic Neuropathies[MeSH Terms]) OR (neuropathy[Title/Abstract])
6	(Incidence[MeSH Terms]) OR (incidence[Title/Abstract]) OR (incident[Title/Abstract]) OR (cohort study[Title/Abstract]) OR (cohort studies[MeSH Terms]) OR (prospective study[Title/Abstract]) OR (retrospective study[Title/Abstract]) OR (longitudinal[Title/Abstract])
7	(cross-sectional study[Title/Abstract]) OR (Clinical trial [Publication Type]) OR (Case Reports[Publication Type]) OR (review[Publication Type]) OR (systematic review[Publication Type]) OR (meta-analysis[Publication Type]) OR (comment[Publication Type]) OR (editorial[Publication Type])
8	1 AND (2 OR 3 OR 4 OR 5) AND 6 NOT 7
9	Filters: in the last 10 years, English

ESM Table 2. Search strategies of cohort studies reporting incidence or prevalence of diabetes-related complications in people with type 2 diabetes across different ethnic or racial groups

Pubmed search using keywords:<br>“ethnicity (includes Hispanic or Latino)” “racial groups (includes American Indian or Alaska Native, Asian, Black or African American, Mexican Americans, Native Hawaiian or Pacific Islander, White)”, “indigenous”, “Native American”, “Maori”, “Indians” ,“multi-ethnic” , “multi-racial” , “diabetes” , “diabetes complications”, “diabetic neuropathy” ,“diabetic kidney disease” ,“albuminuria” , “end-stage kidney disease”, “foot ulcers” ,“lower limb amputations”, “cardiovascular disease”<br><br>Included studies written in English published in years 2010 to 2025 (Except for studies related to the indigenous population, diabetic retinopathy, and diabetic neuropathy, given there are relatively scarce recent publications on multiracial or multiethnic cohorts)<br><br>Article type:<br>clinical study, meta-analysis, RCT, systemic reviews, review articles, observational study

ESM Table 3. Search strategies of genetic studies for diabetes-related complications in type 2 diabetes groups

Item	Search terms in PubMed
#1	"Diabetes Mellitus, Type 2"[MeSH Terms] OR NIDDM[Title/Abstract] OR T2DM[Title/Abstract] OR T2D[Title/Abstract] OR "non insulin depend*"[Title/Abstract] OR "noninsulin depend*"[Title/Abstract] OR noninsulin-depend*[Title/Abstract] OR non-insulindepend*[Title/Abstract] OR ((type 2[Title/Abstract] OR type II[Title/Abstract] OR type2[Title/Abstract] OR type II[Title/Abstract] OR Ketosis-Resistant[Title/Abstract]) AND diabet*[Title/Abstract])
#2	"Genome-Wide Association Study"[MeSH Terms] OR "genome wide association stud*"[Title/Abstract] OR "genome wide association stud*"[Title/Abstract] OR "whole genome association stud*"[Title/Abstract] OR genome wide association analysis[Title/Abstract] OR genome wide association scan[Title/Abstract] OR genome wide association analysis[Title/Abstract] OR genome wide association scan[Title/Abstract] OR genome-wide[Title/Abstract] OR genome-wide[Title/Abstract] OR GWAS[Title/Abstract]
#3	"Genetic Risk Score"[MeSH Terms] OR polygenic score[Title/Abstract] OR polygenetic risk score[Title/Abstract] OR gene score[Title/Abstract] OR genetic score[Title/Abstract]
#4	"Diabetic Nephropathies"[Mesh Terms] OR "chronic kidney disease*"[Title/Abstract] OR "diabetic kidney disease*"[Title/Abstract] OR "end-stage kidney disease*"[Title/Abstract] OR "end-stage renal disease*"[Title/Abstract] OR kidney failure[Title/Abstract] OR albuminuria[Title/Abstract] OR CKD[Title/Abstract] OR ESKD[Title/Abstract] OR ESRD[Title/Abstract]

#5	"Diabetic Retinopathy"[MeSH Terms] OR "retinopathy*"[Title/Abstract]
#6	"Diabetic Neuropathies"[MeSH Terms] OR "neuropathy*"[Title/Abstract]
#7	"Cardiovascular Diseases"[MeSH Terms] OR "cardiovascular disease*"[Title/Abstract] OR "myocardial infarct*"[Title/Abstract] OR "heart infarct*"[Title/Abstract] OR "heart disease*"[Title/Abstract] OR "atheroscleros*"[Title/Abstract] OR "atherosclerotic"[Title/Abstract] OR "myocardial ischemia"[Title/Abstract] OR "myocardial ischaemia"[Title/Abstract] OR "myocardial revascularisation"[Title/Abstract] OR "acute coronary syndrome*"[Title/Abstract] OR "coronary heart disease*"[Title/Abstract] OR "coronary artery disease*"[Title/Abstract] OR "coronary disease*"[Title/Abstract] OR "ischemic heart disease*"[Title/Abstract] OR "ischaemic heart disease*"[Title/Abstract] OR ASCVD[Title/Abstract] OR IHD[Title/Abstract] OR AMI[Title/Abstract] OR ACS[Title/Abstract] OR CHD[Title/Abstract] OR CAD[Title/Abstract] OR "coronary artery bypass*"[Title/Abstract] OR "percutaneous coronary intervention*"[Title/Abstract] OR "percutaneous coronary revascularization"[Title/Abstract] OR "percutaneous coronary revascularisation"[Title/Abstract] OR "percutaneous coronary angioplasty"[Title/Abstract] OR "PCI"[Title/Abstract] OR "major adverse cardiovascular event"[Title/Abstract] OR "major adverse cardiac events"[Title/Abstract] OR MACE[Title/Abstract]
Strategy for GWAS on diabetic complications	#1 AND #2 AND (#4 OR #5 OR #6 OR #7)
Strategy for polygenic	#1 AND #3 AND (#4 OR #5 OR #6 OR #7)

risk scores on diabetic complications	

ESM Table 4. Details of studies reporting incidence rate (per 1,000 person-years) of cardiovascular disease in people with type 2 diabetes across different regions and countries.

Source	Cohort study design	Country	Sample size	Age at enrolment, years	Diabetes duration, years	Outcome definition	Follow-up (FU) years, or person-years (PY)	Number of incident cases	Incidence rate per 1,000 PY
Africa									
Adare, 2024 [61]	Hospital-based cohort	Ethiopia	434	54.7 ± 11.8	1.1 ± 0.8	CAD, stroke and PAD	Median 1.5 years<br>661 PY	177	267.88
Europe									
Thöni, 2023 [1]	Clinic-based cohort	Austria	610	65 (58-71)	7.0 (3.0, 12.0)	MACE	2,752 PY	79	28.71
Olesen, 2022 [2]	Nationwide population-based cohort	Denmark	383,325	59 ± 17	0	Non-fatal myocardial infarction	2,640,815 PY	19,157	7.25
						Non-fatal ischemic stroke		3,352	1.27
						Cardiovascular death		24,267	9.19
						MACE		41,029	15.54
Falkentoft, 2021 [24]	Nationwide population-based cohort	Denmark	57,106	Median 60-61 years	0	Myocardial infarction	155,989 PY	708	4.54
						Stroke		1,058	6.78
						Cardiovascular death		1,021	6.55
						MACE		2,139	13.71
Blin, 2024 [3]	Nationwide population-based cohort	France	1,591,428	65.2 ± 12.1	NR	Myocardial infarction	4.8 ± 0.7	NR	3.9
						Stroke			5.1
						PAD			6.2
Gellen, 2020 [25]	Hospital-based cohort	France	1,321	64 ± 11	12 (6-20)	MACE	7.4 (4.8-10.8)<br>9,965 PY	497	49.9
Rathmann, 2022 [4]	Population-based cohort	Germany	312,368	64.4 ± 13.0	0	Non-fatal stroke or TIA	Mean 4.9 years	16,701	9.3
Polemiti, 2021 [5]	Population-based cohort in Potsdam	Germany	1,083	60.4 (53.5-65.3)	0	Myocardial infarction or stroke	12,517 PY	85	6.79

Thöni, 2023 [1]	Clinic-based cohort	Hungary	1,232	64 (58-70)	12.0 (6.0-19.0)	MACE	5,686 PY	56	9.85
Ballotari, 2017 [6]	Population-based cohort	Italy	24,348	Median 67-70 years	NR	Stroke	68,788 PY	858	12.47
						Myocardial infarction	69,045 PY	700	10.14
						Heart failure	68,965 PY	787	11.41
Harms, 2023 [7]	Population-based cohort	Netherlands	11,993	62.4 ± 12.1	0.6 (0.1-3.2)	CAD, heart failure and sudden cardiac arrest	6.6 (3.1-10.7) 89,470 PY	905	10.12
van Wijingaarden, 2017 [8]	Population-based cohort	Netherlands	32,725	65 (57-73)	NR	CAD	5.4 (2.5-7.8)	2,129	14.0
						Stroke or TIA		1,664	10.6
Jimenez, 2024 [9]	Population-based cohort in Catalonia	Spain	247,751	66.8 ± 11.9	6.3 ± 5.2	CAD, cerebrovascular disease, PAD and heart failure	1,435,568 PY	57,152	39.81
Sattar, 2023 [10]	Nationwide population-based cohort	Sweden	679,072	64.6 ± 12.6	3.9 ± 6.1	Non-fatal CAD	1,120,996 PY	15,875	14.16
						Non-fatal myocardial infarction	1,340,751 PY	8,603	6.42
						Non-fatal stroke	1,291,563 PY	12,297	9.52
						Non-fatal heart failure	1,275,416 PY	21,194	16.62
Höskuldsdóttir, 2022 [11]	Nationwide population-based cohort	Sweden	100,878	62.6 ± 12.5	0	Myocardial infarction, angina pectoris and stroke	Mean 5.5 years	NR	15.9
Li, 2025 [12]	Population-based cohort (UK biobank)	United Kingdom	13,706	Median 56-61 years	Median 3.00-3.43 years	CAD, not including angina pectoris	Median 13 years	2,205	13.26
						Ischemic stroke		570	3.26
						PAD		615	3.52
						Composite		2,927	18.00
Wu, 2024 [26]	Population-based cohort (UK biobank)	United Kingdom	15,118	Mean 56.9-60.6 years	NR	CAD, including angina pectoris	Median 13 years	3,467	19.79
						Stroke		811	4.19
						Heart failure		1,465	7.63
						Composite		4,421	25.85
Khunti, 2022 [13]	Nationwide population-based cohort	United Kingdom	42,869	61 ± 13	3.5 ± 3.6	MACE	Mean 6.6 years	NR	14.0
Middle-East and North Africa									

Faghihi-Kashani, 2016 [64]	Population-based cohort in Tehran	Iran	2,607	Mean 54.1-55.7 years	Mean 4.0-8.3 years	CAD	Median 7.2 years	299	15.87
Zafrir, 2021 [65]	Clinic-based cohort	Israel	735	63.4 ± 5.3	10.1 ± 7.6	MACE	6,730 PY	90	13.37
Farah, 2023 [66]	Hospital-based cohort	Jordan	1,172	59 (53-67)	10.2 ± 7.4	Non-fatal CAD and ischemic stroke	2.9 ± 0.4	147	43.25
Alrawahi, 2018 [67]	Clinic-based cohort	Oman	2,039	54.5 ± 11.4	5.8 ± 4.1	CAD, stroke and PAD	5.3 ± 1.1<br>10,910 PY	192	17.60
North America and Caribbean									
Shah, 2024 [27]	Population-based cohort in Ontario	Canada	25,088	61.4 ± 12.9	Median 2.8 years	Myocardial infarction	Median 6 years	1,044	6.65
						Stroke		581	3.66
						Cardiovascular death		439	3.35
An, 2021 [28]	Population-based cohort in Southern California	United States	135,199	57.8 ± 13.2	0	Myocardial infarction	Median 5.9 years	3,519	4.2
						Unstable angina		2,597	3.1
						Stroke		5,578	6.6
						Composite of above		9,645	11.9
						PAD		3,967	4.8
Liu, 2020 [30]	Population-based cohort	United States	10,809	Mean 59.2-66.7 years	0	Fatal and non-fatal CAD and stroke	153,166 PY	2,580	16.84
South and Central America									
Cardoso, 2025 [71]	Hospital-based cohort	Brazil	685	60.0 ± 9.5	8 (range 3 to 15)	MACE	10.7 (6.3-13.2)<br>6,606 PY	173	27.5
South-East Asia									
Anjana, 2015 [73]	Hospital-based cohort	India	3,581	Mean 52.4-55.5 years	Mean 11.1-18.3 years	CAD	10,892 PY	74	6.79
						PAD	10,701 PY	36	3.36
Western Pacific									
Morton, 2022 [31]	Nationwide population-based cohort	Australia	1,091,066	At end of FU: 70.2 (60.2-79.3)	At end of FU: 10.3 (5.4-16.7)	Myocardial infarction	6,906,749 PY	57,813	8.37
						Stroke		40,069	5.80
Sun, 2022 [32]	Population-based cohort	China	26,004	57.4 ± 9.6	NR	MACE	Median 10.2 years<br>265,700 PY	6,848	25.77

Li, 2024 [33]	Population-based cohort in Tangshan	China	19,915	55.3 ± 10.7	NR	Stroke	Median 11.5 years<br>204,317 PY	1,776	8.69
Zhang, 2019 [57]	Population-based cohort in Shanghai	China	1,880	59.1 ± 6.9	6.0 (2.0-9.0)	CAD and stroke	5,449 PY	100	18.35
Fan, 2023 [34]	Territory-wide population-based cohort	Hong Kong	499,288	61.8 ± 11.9	2.0 (0.0-7.0)	CAD	7.5 ± 4.3	25,769	7.40
						Myocardial infarction		15,483	4.10
						Stroke		24,497	6.90
						PAD		7,668	2.03
						CAD, stroke and PAD		44,463	14.02
Kadowaki, 2022 [35]	Population-based cohort	Japan	426,186	66.5 ± 12.4	NR	Myocardial infarction	2.6 ± 2.0	2,304	2.1
						Stroke		12,002	11.1
						PAD		6,118	5.6
Moon, 2023 [36]	Population-based cohort	Korea	248,002	59.6 ± 10.9	NR	Myocardial infarction	9.3 (9.0-9.6)	NR	3.49
						Ischemic stroke			7.07
						Composite			10.19
Choi, 2020	Population-based cohort	Korea	36,058	≥ 40	0	CAD	7.0 ± 2.5<br>253,062 PY	4,138	16.35
						Stroke		2,759	10.90
						Composite		6,897	27.25
Yu, 2023 [37]	Population-based cohort	New Zealand	36,267	55.4 ± 13.5	4.8 ± 1.2	Myocardial infarction	489,578 PY	4,786	9.78
						Stroke	512,001 PY	3,016	5.89
Seng, 2020 [38]	Population-based cohort	Singapore	71,125	64.0 ± 12.6	4.9 ± 3.1	Myocardial infarction	4 years	3,157	11.10
						Stroke		1,931	6.79
Chou, 2021 [39]	Nationwide population-based cohort	Taiwan	115,751	60.1 ± 12.2	0	MACE	850,771 PY	30,305	35.62

Data presented as mean ± standard deviation (SD) or median (interquartile range, IQR). CAD, coronary arterial disease; MACE, major adverse cardiovascular events, including non-fatal myocardial infarction, stroke and cardiovascular death; NR, not reported; PAD, peripheral arterial disease; TIA, transient ischemic attack.

ESM Table 5. Details of studies reporting incidence rate (per 1,000 person-years) of chronic kidney disease and end-stage kidney disease in people with type 2 diabetes across different regions and countries.

Source	Cohort study design	Country	Sample size	Age at enrolment, years	Diabetes duration, years	Outcome definition	Follow-up (FU) years, or person-years (PY)	Number of incident cases	Incidence rate per 1,000 PY
**Chronic kidney disease**									
Africa									
Tamru, 2020 [62]	Hospital-based cohort	Ethiopia	346	56.7 ± 10.5	0	eGFR <60 mL/min/1.73 m^2	1,877 PY	NR	36.0
Europe									
Thöni, 2023 [1]	Clinic-based cohort	Austria	610	65 (58-71)	7.0 (3.0-12.0)	Sustained eGFR decline ≥ 40%, sustained albuminuria increase ≥ 30%, progression to KRT, or death from renal cause	2,712 PY	73	26.92
Scheuer, 2022 [14]	Nationwide population-based cohort	Denmark	371,625	63 ± 14	0	Moderate, severe and end-stage kidney disease	2,570,520 PY	83,560	32.51
Blin, 2024 [3]	Nationwide population-based cohort	France	1,591,428	65.2 ± 12.1	NR	ICD-10 codes for renal failure, diabetes and hypertension related renal disease	4.8 ± 0.7	NR	17.9
Polemiti, 2021 [5]	Population-based cohort in Potsdam	Germany	1,083	60.4 (53.5-65.3)	0	DKD, albuminuria or KRT	12,608 PY	207	16.42
Thöni, 2023 [1]	Clinic-based cohort	Hungary	1,232	64 (58-70)	12.0 (6.0-19.0)	Sustained eGFR decline ≥ 40%, sustained albuminuria increase ≥ 30%, progression to KRT, or death from renal cause	5,324 PY	171	32.12
Russo, 2016 [15]	Population-based cohort	Italy	15,362	64 ± 9	10 ± 8	Albuminuria	4 years	3,570	58.10
						eGFR <60 mL/min/1.73 m^2		1,962	31.93

van Wijingaarden, 2017 [8]	Population-based cohort	Netherlands	32,725	65 (57-73)	NR	eGFR <60 mL/min/1.73 m^2, or albuminuria >30 mg/L, or diagnosis of CKD	5.4 (2.5-7.8) 124,708 PY	7,507	60.2
Salinero-Fort, 2015 [16]	Multi-centre cohort	Spain	2,620	67.3 ± 10.8	8.5 ± 7.4	eGFR <60 mL/min/1.73 m^2	Median 5 years	NR	24.8
Höskuldsdóttir, 2022 [11]	Nationwide population-based cohort	Sweden	100,878	62.6 ± 12.5	0	ICD-9 and ICD-10 codes for DKD and CKD	Mean 5.5 years	NR	5.3
Li, 2025 [12]	Population-based cohort	United Kingdom	13,706	Median 56-61 years	Median 3.00-3.43 years	eGFR <60 mL/min/1.73 m^2, DKD and CKD	Median 13 years	1,114	6.42
Middle-East and North Africa									
Farah, 2023 [66]	Hospital-based cohort	Jordan	1,172	59 (53-67)	10.2 ± 7.4	eGFR <30 mL/min/1.73 m^2	2.9 ± 0.4	61	17.95
North America and Caribbean									
An, 2021 [28]	Population-based cohort in Southern California	United States	135,199	57.8 ± 13.2	0	Two or more eGFR <60 mL/min/1.73 m^2 within 90 days	Median 5.3 years	14,609	21.2
South and Central America									
Cardoso, 2025 [71]	Hospital-based cohort	Brazil	586	60.0 ± 9.5	8 (range 3 to 15)	New microalbuminuria development or progression to macroalbuminuria	10.7 (6.3-13.2) 6,606 PY	127	25.5
			685			Doubling of serum creatinine, progression to KRT, or death from renal cause		104	16.9
Carrasco-Tenezaca, 2022 [72]	Clinic-based cohort	Ecuador	513	65.7 ± 11.7	13.3 ± 7.2	eGFR <60 mL/min/1.73 m^2 for more than 3 months	10 years	207	32.07
South-East Asia									
Anjana, 2015 [73]	Hospital-based cohort	India	3,581	Mean 52.4-55.5 years	Mean 11.1-18.3 years	Albuminuria (Urinary ACR ≥ 30 mg/g)	25,312 PY	891	35.20
Fawwad, 2018 [74]	Hospital-based cohort	Pakistan	4,633	50.7 ± 10.8	NR	Albuminuria (criteria not specified)	10,769 PY	1,144	106.2

Western Pacific									
Chen, 2024 [45]	Hospital-based cohort	China	1,436	64.3 ± 7.5	NR	eGFR <60 mL/min/1.73 m^2 or albuminuria >20 mg/L	Median 2.4 years<br>3478 PY	109	31.34
Wu, 2023 [40]	Territory-wide population-based cohort	Hong Kong	102,488	66 (54-76)	NR	ICD-9 codes for CKD	7.5 (3.5-11.5)	14,798	17.6
Wu, 2021 [58]	Territory-wide population-based cohort	Hong Kong	436,744	59.7 ± 11.8	0	eGFR <60 mL/min/1.73 m^2, or ICD-9 codes for kidney dialysis and transplant	Median 5.3 years<br>2,573,633 PY	134,043	52.1
Chen, 2023 [41]	Nationwide population-based cohort	Taiwan	113,449	59.6 ± 13.6	5.7 ± 5.4	ICD-9 and ICD-10 codes for CKD	139,602 PY	2,804	20.09
Kadowaki, 2022 [35]	Population-based cohort	Japan	426,186	66.5 ± 12.4	NR	ICD-10 codes for renal failure, diabetes and hypertension related renal disease	2.6 ± 2.0	17,002	15.8
Jang, 2024 [42]	Nationwide population-based cohort	Korea	212,836	Mean 48-50.2 years	0	eGFR <60 mL/min/1.73 m^2, or ICD-10 codes for CKD	5.23 ± 1.75<br>1,113,727 PY	6,850	6.15
**End-stage kidney disease**									
Europe									
Finne, 2019 [17]	Nationwide population-based cohort	Finland	421,429	≥ 40 years	NR	Progression to KRT	Median 6.82 years<br>3,458,797 PY	1,516	0.44
North America and Caribbean									
Shah, 2024 [27]	Population-based cohort	Canada	25,088	61.4 ± 12.9	Median 2.8 years	Progression to KRT	Median 6 years	136	0.85
An, 2021 [28]	Population-based cohort in Southern California	United States	135,199	57.8 ± 13.2	0	Progression to KRT	Median 5.9 years	870	1.0
Western Pacific									

Morton, 2022 [31]	Population-based cohort	Australia	1,091,066	At end of FU: 70.2 (60.2-79.3)	At end of FU: 10.3 (5.4-16.7)	Progression to KRT, or death from renal cause	6,906,749 PY	13,331	1.93
Fan, 2023 [34]	Territory-wide population-based cohort	Hong Kong	499,288	61.8 ± 11.9	2.0 (0.0-7.0)	Two eGFR <15 mL/min/1.73 m^2 or progression to KRT	7.6 ± 4.3 3,799,147 PY	20,120	5.30
Iwase, 2022 [43]	Multi-centre cohort	Japan	4,904	65 ± 10	16 ± 11	Progression to KRT	Median 5.3 years	104	4.1
Jung, 2024 [44]	Population-based cohort	Korea	847,884	Mean 56.4-66.1 years	NR	eGFR <15 mL/min/1.73 m^2 or progression to KRT	7,693,881 PY	11,825	1.54
Yu, 2023 [37]	Population-based cohort	New Zealand	36,267	55.4 ± 13.5	4.8 ± 1.2	ICD-9 and ICD-10 codes (did not list detailed codes)	483,001 PY	7,486	15.50 (9.4 in Asian and 20.9 in Māori)
Seng, 2020 [38]	Population-based cohort	Singapore	71,125	64.0 ± 12.6	4.9 ± 3.1	Progression to KRT	4 years	1,251	4.40

Data presented as mean ± standard deviation (SD) or median (interquartile range, IQR). CKD, chronic kidney disease; DKD, diabetic kidney disease; eGFR, estimated glomerular filtration rate; KRT, kidney replacement therapy; NR, not reported

ESM Table 6. Details of studies reporting incidence rate (per 1,000 person-years) of diabetic retinopathy across different regions and countries

Source	Cohort study design	Country	Sample size	Age at enrolment, years	Diabetes duration, years	Outcome definition	Follow-up (FU) years, or person-years (PY)	Number of incident cases	Incidence rate per 1,000 PY
Africa									
Kebede, 2022 [63]	Hospital-based cohort	Ethiopia	466	53.2 ± 10.1	9.2 ± 3.8	Fundus examination	Median 7.6 years<br>3,608 PY	80	20
Europe									
Scheuer, 2022 [14]	Nationwide population-based cohort	Denmark	371,625	63 ± 14	0	Potentially treatment requiring moderate and severe retinopathy	2,737,247 PY	17,246	6.30
van Wijingaarden, 2017 [8]	Population-based cohort	Netherlands	32,725	65 (57-73)	NR	Record of retinopathy, maculopathy or diabetic macular oedema in database	5.4 (2.5-7.8)	3,764	25.0
Hernández-Teixidó, 2024 [18]	Population-based cohort in Catalonia	Spain	146,506	65.4 ± 11.2	5.7 ± 5.1	Retinal photography graded based on ICDR Severity Scale	Median 6.96 years	6,881	6.99
Li, 2025 [12]	Population-based cohort	United Kingdom	13,706	Median 56-61 years	Median 3.00-3.43 years	ICD-10 codes for diabetic retinopathy	Median 13 years	1,393	8.10
Middle-East and North Africa									
Dehghani Firouzabadi, 2024 [68]	Hospital-based cohort	Iran	1,145	Mean 61.4-62.7 years	Mean 15.3-20.0 years	ICD-10 codes for diabetic retinopathy	10 years	294	25.68
North America and Caribbean									
Shah, 2024 [27]	Population-based cohort in Ontario	Canada	24,476	61.4 ± 12.9	Median 2.8 years	Retinopathy treatment: claims from an ophthalmologist with related fee codes	Median 6 years	807	5.26
An, 2021 [28]	Population-based cohort in	United States	135,199	57.8 ± 13.2	0	ICD-9 and ICD-10 codes for proliferative diabetic retinopathy	Median 5.9 years	1,353	1.6

	Southern California								
South and Central America									
Cardoso, 2025 [71]	Hospital-based cohort	Brazil	547	60.0 ± 9.5	8 (range 3 to 15)	Annual ophthalmologic examination for retinopathy development or worsening	10.7 (6.3-13.2)<br>6,606 PY	160	50.6
South-East Asia									
Anjana, 2015 [73]	Hospital-based cohort	India	3,581	Mean 52.4-55.5 years	Mean 11.1-18.3 years	Fundus examination by retinal specialist annually	17,254 PY	632	36.63
Fawwad, 2018 [74]	Hospital-based cohort	Pakistan	4,633	50.7 ± 10.8	NR	Direct fundoscopy founded pre-proliferative and proliferative retinopathy, macular oedema or diabetes-related blindness	2,379 PY	221	92.8
Western Pacific									
Sun, 2022 [32]	Population-based cohort	China	26,004	57.4 ± 9.6	NR	ICD-10 codes for diabetic retinopathy	Median 10.2 years<br>265,700 PY	781	2.94
Chen, 2024 [45]	Hospital-based cohort	China	1,436	64.3 ± 7.5	NR	Fundus photography interpreted by specialists annually	Median 2.4 years<br>3,478 PY	277	79.64
Kuwata, 2017 [59]	Hospital-based cohort	Japan	1,814	65.5 ± 11.5	11.4 ± 8.6	Fundus examination conducted by ophthalmologists	3,390 PY	184	54.3
Fukuda, 2017 [50]	Claims-based cohort	Japan	11,331	Mean 50.0-52.3 years	NR	ICD-10 codes for diabetic retinopathy	Mean 2.2 years	444	17.7
Huh, 2023 [46]	Nationwide population-based cohort	Korea	399,984	59.9 ± 8.7	NR	ICD-10 codes for proliferative retinopathy along with related treatment	7.6 ± 1.8	20,054	6.60
Tham, 2020 [47]	Population-based cohort	Singapore	972 (1,734 eyes)	58.7 ± 9.1	Mean 5.2-6.6 years	Fundus examination	6.3 ± 0.9	272 eyes	24.90
Jeng, 2022 [48]	Population-based cohort	Taiwan	73,964	57.2 ± 13.8	0	ICD-9 codes for diabetic retinopathy, diabetic macular edema and related treatment	440,908 PY	5,845	13.26

ESM Table 7. Details of studies reporting incidence rate (per 1,000 person-years) of diabetic neuropathy and non-traumatic lower limb extremity amputation in people with type 2 diabetes across different regions and countries

Source	Cohort study design	Country	Sample size	Age at enrolment, years	Diabetes duration, years	Outcome definition	Follow-up (FU) years, or person-years (PY)	Number of incident cases	Incidence rate per 1,000 PY
**Diabetic neuropathy**									
Africa									
Tantigegn, 2023 [69]	Hospital-based cohort	Ethiopia	669	54.8 ± 10.9	0	Related symptoms or indications by examination	10.3 (7.2-12.4) 6,433 PY	138	21.4
Europe									
Kristensen, 2020 [19]	Nationwide population-based cohort	Denmark	259,625	62 (52-72)	0	Hospital diagnosis codes for diabetic polyneuropathy	6.21 (3.39-9.57)	6,677	3.85
Coman, 2024 [20]	Population-based cohort	Romania	NR	NR	NR	ICD-10 codes for diabetic polyneuropathy	NR	NR	3.07
Li, 2025 [12]	Population-based cohort	United Kingdom	13,706	Median 56-61 years	Median 3.00-3.43 years	ICD-10 codes for diabetes with neurological complications, diabetic mononeuropathy, polyneuropathy, autonomic neuropathy	Median 13 years	582	3.33
Middle-East and North Africa									
Firouzabadi, 2023 [70]	Hospital-based cohort	Iran	1,152	Mean 51.4-52.4 years	Mean 5.6-8.7 years	ICD-10 codes for diabetes with neurological complications	10 years	334	28.99
North America and Caribbean									
An, 2021 [28]	Population-based cohort in Southern California	United States	135,199	57.8 ± 13.2	0	ICD-9 codes for diabetes with neurological complications, diabetic mononeuropathy, polyneuropathy, autonomic neuropathy	Median 5.2 years	19,957	26.9
South and Central America									

Cardoso, 2025 [71]	Hospital-based cohort	Brazil	519	60.0 ± 9.5	8 (3 to 15)	Neuropathy development or worsening by annual examination	10.7 (6.3-13.2)<br>6,606 PY	178	42.7
South-East Asia									
Anjana, 2015 [73]	Hospital-based cohort	India	3,581	Mean 52.4-55.5 years	Mean 11.1-18.3 years	Vibratory perception threshold ≥20 V	11,561 PY	484	41.86
Fawwad, 2018 [74]	Hospital-based cohort	Pakistan	4,633	50.7 ± 10.8	NR	Abnormality in pin prick, vibration sense, ankle and knee reflex	6,138 PY	799	130.2
Western Pacific									
Davis, 2024 [49]	Clinic-based cohort	Australia	1,509	Mean 65 years	Median 8.0 years	Vibratory perception threshold ≥20 V	2,399 PY	177	73.78
Sun, 2022 [32]	Population-based cohort	China	26,004	57.4 ± 9.6	NR	ICD-10 codes for diabetes with neurological complications	Median 10.2 years<br>265,700 PY	888	3.34
Fukuda, 2017 [50]	Claims-based cohort	Japan	11,331	Mean 50.0-52.3 years	NR	ICD-10 codes for diabetes with neurological complications, diabetic mononeuropathy, polyneuropathy, autonomic neuropathy	Mean 2.2 years	98	3.91
Pek, 2020 [51]	Clinic-based cohort	Singapore	1,250	Mean 56.3-59.6 years	Mean 10.1-13.9 years	Abnormal findings in monofilament or vibratory perception threshold ≥25 V	2.9 ± 0.7	134	36.97
Yang, 2017 [52]	Population-based cohort	Taiwan	36,152	Mean 59.6-60.3 years	Mean 5.1-5.8 years	ICD-9 codes for diabetic polyneuropathy	Mean 7.23 years	7,219	27.62
**Non-traumatic limb amputation**									
Europe									
Potier, 2019 [21]	Hospital-based cohort	France	1,459	Mean 62.9-67.1 years	Mean 13.1-16.2 years	Amputation at or above the metatarsophalangeal joint	Median 7.1 years	79	7.8
Lopez-de-Andres, 2022 [22]	Population-based cohort	Spain	NR	NR	NR	Minor amputation (below the ankle joint)	2001-2019	77,082	2.07
						Major amputation (above the ankle joint)		51,977	1.40
			83,688	68.0 ± 12.5	7.4 ± 5.5	Minor and major amputation	3.9 ± 2.0	521	1.62

Gunn, 2021 [23]	Population-based cohort	United Kingdom				Major-only amputation		309	0.96
North America and Caribbean									
Shah, 2024 [27]	Population-based cohort in Ontario	Canada	25,088	61.4 ± 12.9	Median 2.8 years	Procedure for limb amputation	Median 6 years	191	1.20
Déruaz-Luyet, 2020 [29]	Claims-based cohort	United State	2,300,411	58.8 ± 12.4	NR	Minor amputation	Mean 2.5 years	7,610	1.34
						Major amputation		2,297	0.40
						Composite of above		9,222	1.62
Western Pacific									
Morton, 2022 [31]	Nationwide population-based cohort	Australia	1,091,066	At end of FU: 70.2 (60.2, 79.3)	At end of FU: 10.3 (5.4, 16.7)	Major and minor amputation	6,906,749 PY	13,234	1.92
Iwase, 2018 [53]	Registry-based cohort	Japan	4,870	Mean 65.4-67.1 years	Mean 15.5-19.5 years	Procedure for limb amputation	Median 5.3 years	12	0.47
Huh, 2023 [46]	Nationwide population-based cohort	Korea	399,984	59.9 ± 8.7	NR	Procedure for limb amputation	7.6 ± 1.8	1,943	0.64
Robinson, 2016 [54]	Population-based cohort	New Zealand	62,002	Mean 55-66 years	Median 3-4 years	Procedure for limb amputation	Median 7.14 years	892	2.11 (0.29 in East Asian and 3.48 in Maori)
Seng, 2020 [38]	Population-based cohort	Singapore	71,125	64.0 ± 12.6	4.9 ± 3.1	NR	4 years	664	2.33
Hsiao, 2023 [55]	Population-based cohort	Taiwan	56,872	64.0 ± 11.6	NR	Major amputation	6.1 ± 2.1	NR	1.25
Li, 2020 [56]	Population-based cohort	Taiwan	27,574	Mean 64.5-68.5 years	Mean 7.3-10.7 years	Minor amputation	Median 8.9 years	310	1.3 in women and 1.8 in men
						Major amputation		195	0.9 in women and 1.2 in men
						Composite of above		541	2.4
