# Supplementary material for: Heterogeneity in the development of diabetes-related complications: narrative review of the roles of ancestry and geographical determinants
Source: Diabetologia. 2025 Jul 22;68(11):2386–404. doi: 10.1007/s00125-025-06482-8 (PMC12534336; doi:10.1007/s00125-025-06482-8)
Supplement: Supplementary file 2 — Slideset of figures (PPTX 313 KB) [file 125_2025_6482_MOESM2_ESM.pptx]

## Slide 1
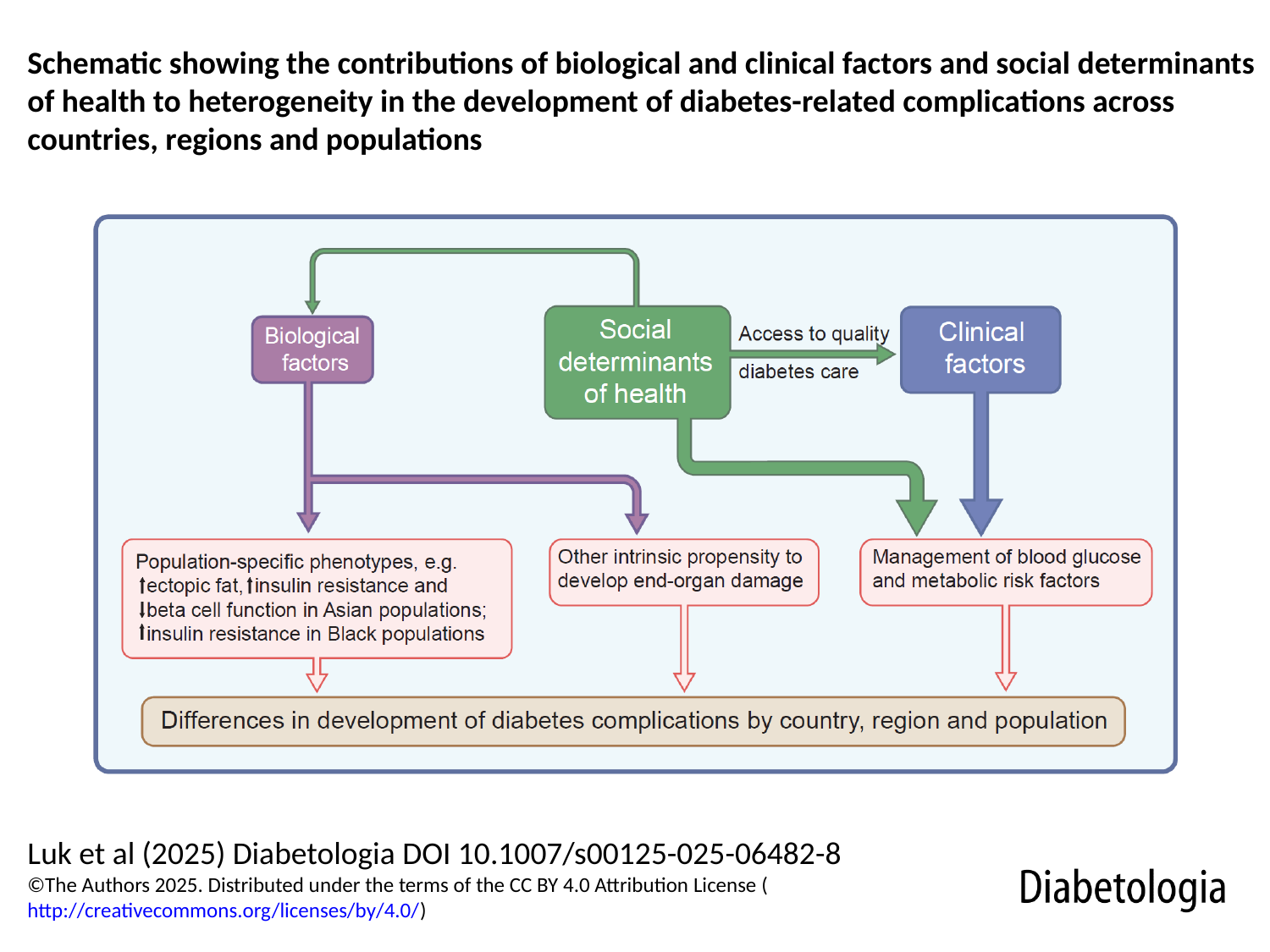

Schematic showing the contributions of biological and clinical factors and social determinants of health to heterogeneity in the development of diabetes-related complications across countries, regions and populations
Luk et al (2025) Diabetologia DOI 10.1007/s00125-025-06482-8
©The Authors 2025. Distributed under the terms of the CC BY 4.0 Attribution License (http://creativecommons.org/licenses/by/4.0/)

## Slide 2
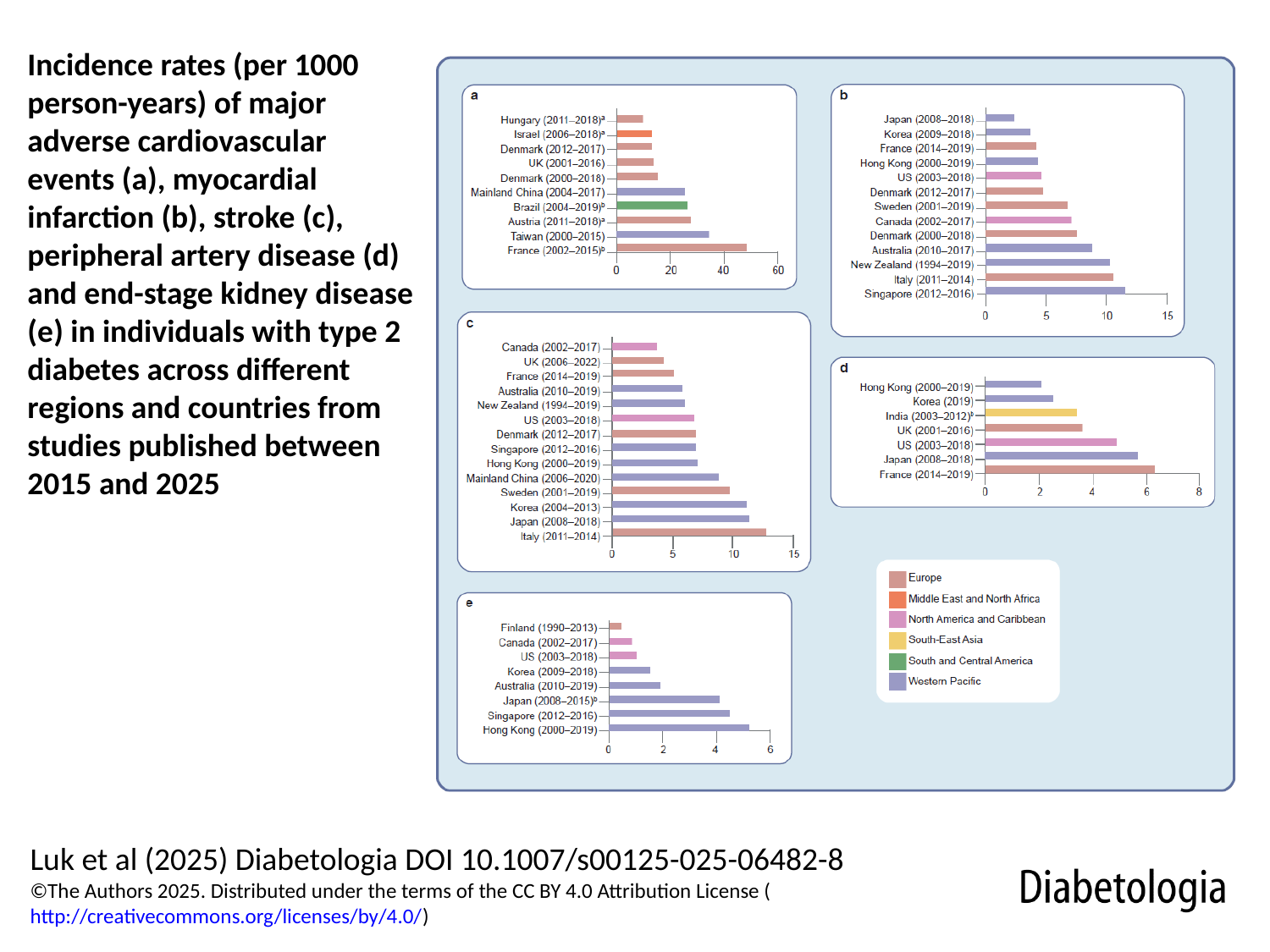

Incidence rates (per 1000 person-years) of major adverse cardiovascular
events (a), myocardial infarction (b), stroke (c), peripheral artery disease (d) and end-stage kidney disease (e) in individuals with type 2 diabetes across different regions and countries from studies published between 2015 and 2025
Luk et al (2025) Diabetologia DOI 10.1007/s00125-025-06482-8
©The Authors 2025. Distributed under the terms of the CC BY 4.0 Attribution License (http://creativecommons.org/licenses/by/4.0/)
